# A high molecular weight hyaluronic acid biphasic dispersion as potential therapeutics for interstitial cystitis

**DOI:** 10.1002/jbm.b.34751

**Published:** 2020-10-26

**Authors:** Peadar R. Rooney, Vijaya Krishna Kannala, Niranjan G. Kotla, Ana Benito, Damien Dupin, Iraida Loinaz, Leo R. Quinlan, Yury Rochev, Abhay Pandit

**Affiliations:** ^1^ CÚRAM, SFI Research Centre for Medical Devices National University of Ireland Galway Galway Ireland; ^2^ CIDETEC Parque Científicoy Tecnológico de Gipuzkoa San Sebastián Spain; ^3^ Physiology, School of Medicine National University of Ireland Galway Galway Ireland; ^4^ Sechenov First Moscow State Medical University Institute for Regenerative Medicine Moscow Russian Federation

**Keywords:** barrier effect, hyaluronic acid, inflammation, interstitial cystitis, permeability

## Abstract

Interstitial cystitis (IC) is a progressive bladder disease characterized by increased urothelial permeability, inflammation of the bladder with abdominal pain. While there is no consensus on the etiology of the disease, it was believed that restoring the barrier between urinary solutes and (GAG) urothelium would interrupt the progression of this disease. Currently, several treatment options include intravesical delivery of hyaluronic acid (HA) and/or chondroitin sulfate solutions, through a catheter to restore the urothelial barrier, but have shown limited success in preclinical, clinical trials. Herein we report for the first time successful engineering and characterization of biphasic system developed by combining cross‐linked hyaluronic acid and naïve HA solution to decrease inflammation and permeability in an in vitro model of interstitial cystitis. The cross‐linking of HA was performed by 4‐arm‐polyethyeleneamine chemistry. The HA formulations were tested for their viscoelastic properties and the effects on cell metabolism, inflammatory markers, and permeability. Our study demonstrates the therapeutic effects of different ratios of the biphasic system and reports their ability to increase the barrier effect by decreasing the permeability and alteration of cell metabolism with respect to relative controls. Restoring the barrier by using biphasic system of HA therapy may be a promising approach to IC.

## 1 INTRODUCTION

Interstitial cystitis (IC) is a chronic disease of the bladder, characterized by bladder pain, increased urinary frequency and urgency, nocturia, and significant lifestyle problems.^1^, ^2^, ^3^, ^4^ The etiology of IC remains unclear, but various hypotheses including autoimmune processes, allergic reactions, chronic bacterial infections, toxic or dietary exposure, and psychosomatic factors were considered as key players.^5^ The bladder includes a specialized epithelial layer composed of urothelial cells that form a tight, impermeable, protective barrier made up of glycosaminoglycan (GAG) and sulfated glycosaminoglycan (sGAG).^6^, ^7^, ^8^, ^9^ Disruption of this bladder wall leads to increased permeability of the bladder, which is a common clinical event in all patients with IC.^10^ One of the most common IC treatment is intravesical sGAG or GAGs instillation into the bladder through a catheter. These treatments are targeted to augment a major protective element of the bladder, the urothelial lining and provide permissive healing environment for the urothelium.^11^, ^12^, ^13^, ^14^, ^15^ Most of intravesical sGAG/GAG treatments are composed of hyaluronan (HA) solutions either alone or in combination with other GAGs (such as chondroitin sulfate).^11^ HA instillations have been shown to decrease secretion of inflammatory cytokines and have been identified as a potential cost‐effective treatment for IC patients.^16^, ^17^, ^18^, ^19^ Furthermore, in several nonblinded, nonplacebo controlled clinical trials; HA has demonstrated a positive patient response with decreased visual analogue scores for pain symptoms.^20^, ^21^, ^22^ However, the clinical formulations use non‐crosslinked HA systems that do not recover the urothelium's permeability, which is one of the main clinical symptoms of IC.

The cross‐linking chemistry of HA and modified HA derivatives are well described in the literature.^23^ It is well accepted that cross‐linking of HA can increase chain length and can decrease macromolecular diffusion in the surrounding.^5^ HA is recognized as suitable for carrier for cells,^24^, ^25^, ^26^, ^27^, ^28^, ^29^ treatment of osteoarthritis^30^ and in colon targeted drug delivery applications^31^ but to the best of our knowledge few studies report on the effect of cross‐linked HA on urothelium specific to IC. HA is degraded mainly by the intestinal bacteria hyaluronidases^32^, ^33^, ^34^, ^35^ and the absence of the bladder enzyme hyaluronidases^35^, ^36^, ^37^, ^38^, ^39^, ^40^, ^41^ it can be expected that long‐chain cross‐linked HA delivered locally via the catheter will remain on the surface of the bladder lumen for prolonged time with additive therapeutic benefits.

Considering this, the manuscript describes an in vitro model of urothelium barrier damage in order to examine the effects of native high molecular weight HA solution (commercial), cross‐linked HA solution (cHA) and the combination of biphasic solution (native HA solution and cHA). HA cross‐linking was carried out as per our reported GMP translatable methodology for the synthesis of cHA.^42^ The cross‐linking was performed with 4‐arm‐polyethyeleneamine (PEG) linker using 4‐(4,6‐Dimethoxy‐1,3,5‐triazin‐2‐yl)‐4‐methylmorpholinium chloride (DMTM) chemistry. The developed HA solutions were subjected to rheological, morphological, cellular level inflammatory cytokine, and GAG assessments. Finally, the HA, cHA, and the biphasic system were studied for concentration dependent effects of the different ratios of HA, cHA, and the biphasic system on the inflammatory cytokines and permeability in the in vitro model to examine their effect on inflammatory cytokines and trans‐epithelial permeability using transwell assay units.

## 2 MATERIAL AND METHODS

### 2.1 Materials

HA (high molecular weight sodium hyaluronate 1.2 X 10^6^ Da), CAS No: 9067‐32‐7 purchased from Lifecore Biomedical, USA. Phosphate buffered saline (Lot # SLBP8218V) purchased from Sigma–Aldrich, USA. PEG (Mw 2000 Da‐CAS No.: 25322–68‐3, purity >95%) purchased from JenKem Technology, USA (Allen, TX). DMTMM (4‐[4.6‐Dimethoxy‐1,3,5‐triazin‐2‐yl]‐4‐methylmorpholinium chloride; Mw 276.72), CAS No: 3945‐69‐5. Na_2_SO_4_ (CAS No. 7757‐82‐6), EtOH (CAS No. 64–17‐5), NaCl (CAS No. 7647‐14‐5), dialysis membrane (Spectra Por, Mol 6–8 kDa), and 2‐(N‐morpholino) ethanesulfonic acid (MES salt) (CAS No. 4432‐31‐9) purchased from Sigma–Aldrich (Arklow, Ireland). HTB‐2 and T84 cells were purchased from ATCC (Manassas, VA). Cell culture reagents, Dulbecco's Modified Eagle's Medium (DMEM) (D5796) Fetal bovine solution (FBS) (F0804), penicillin–streptomycin (P4333), trypsin–EDTA (T4049), Hank balanced salt solution (HBSS) (H8264), protamine sulfate (P3369) and 0.9% NaCl solution (S8776) were purchased from Sigma–Aldrich (Arklow, Ireland). Iron‐enriched FBS for culturing T84 cells (SV30160.03) was purchased from Biosciences (Dublin, Ireland). Recombinant human TNF‐alpha (300‐01A) was purchased from Pepro Tech (London, UK).

### 2.2 Preparation of HA solutions

Different concentrations of naïve HA (1, 3, 9, 15 mg/ml) were prepared by dissolving naïve HA in PBS buffer (pH 6.0) and stirring overnight on a magnetic stirrer at 500 rpm, 25°C.

### 2.3 Synthesis of cHA

The fabricated HA system is a two‐component biphasic dispersion consisting of cHA and non‐cross‐linked HA solutions (naïve HA). cHA was synthesized as per our reported cross‐linking methodology developed for its GMP translation. This methodology is an improvement of our previously reported and the literature procedures for cross‐linking HA.^42^, ^43^, ^44^, ^45^, ^46^ A homogeneous solution of HA (2 g at 10 mg/ml) was prepared in MES buffer (pH 6.0; 0.1 M) at 25°C. Na_2_SO_4_ (20 wt%, 200 ml) was added and stirred (500 rpm, 25°C) until a clear and completely homogeneous solution was observed. DMTMM, as solid (1.46 g, 1 eq. wrt. the HA repeating unit), was added and stirred (500 rpm, 25°C) for 15–20 min. PEG (1 g, 0.4 eq. wrt. HA repeating unit) was predissolved in MES buffer (100 ml) was then added to the reaction mixture to initiate cross‐linking and stirred (500 rpm, 25°C) overnight. PEG (1 g, 0.4 eq. wrt. HA repeating unit) was added to target 40% theoretical cross‐linking, while 1 eq. wrt. HA repeating unit of DMTMM was added to facilitate efficient cross‐linking. Upon completion of the reaction, the mixture was resuspended in a solution of NaCl (8 wt%, twice the volume of reaction mixture) and precipitated by the addition of ethanol (four times the volume of reaction mixture). The precipitate recovered by centrifugation was subsequently washed with 80% aqueous ethanol (once) and 33% aqueous ethanol (twice) and then freeze dried at −80°C to obtain the cross‐linked HA.

### 2.4 Determining degree of cross‐linking of cHA

NMR studies and carbazole assay were performed to determine the degree of cross‐linking of cHA. In NMR, the peak integrals were compared for quantitative estimation. Carbazole assay was carried out following previously reported procedures using samples with and without salts.^42^, ^47^, ^48^ All the reagents were freshly prepared and no commercial kits were used.

### 2.5 Preparation of biphasic dispersion of cHA in HA solution

The cHA and HA ratios were formulated 24–48 hrs before each independent experiment. The cHA was dispersed at 6 mg/ml by adding sterile H_2_O with enough NaCl to bring the final concentration of NaCl to 0.9%. HA was dispersed at 6 mg/ml by adding sterile NaCl and H_2_O to bring the final concentration of NaCl to 0.9%. The ratios of cHA and HA were added to a vial with sterile 0.9% NaCl solution added to dilute the concentration to 1 and 3 mg/ml which was mixed for 24–48 hrs.

### 2.6 Morphological assessment of HA solutions and cHA solutions

The morphology of the HA concentration‐based solutions and cross‐linked HA were analyzed by scanning electron microscopy (SEM). Lyophilized HA solution sample was placed onto a glass cover slip mounted on a sticky carbon pad and gold‐coated high vacuum evaporator (Emitech K550 Sputter Coater) and assessed for surface morphological characteristics (S‐4700‐Hitachi).

### 2.7 Rheological assessment of HA solutions

Rheological measurements of HA solutions were performed with a Discovery Series Rheometer (DHR‐3, TA Instruments, USA), using parallel plate geometry of 60 mm diameter, the selected geometry was chosen to provide a balance between sensitivity and sample volume. HA solutions (1, 3, 9, 15 mg/ml) were vortexed and then each sample was directly loaded on the bottom plate, the upper plate was then lowered to a measurement gap of 500 μm. The measurement parameters were determined to be within the linear viscoelastic region in preliminary experiments by amplitude and frequency sweeps. The measurement was allowed to proceed until the storage modulus (G′) and the loss modulus (G″) reached a plateau. The modulus (G′, G″) and complex viscosity (Pa.s) were taken at 37°C in the dynamic oscillatory mode with amplitude sweep (0.1–10% strain at 1 Hz frequency) and time sweep (at 1 Pa stress, 0.1 Hz frequency for 5 min).

### 2.8 Cell culture studies

#### 2.8.1 HTB‐2 cell experimental conditions

Human urothelial cells HTB2 were grown on tissue culture plastic in basal media consisting of DMEM supplemented with 10% fetal calf serum (FCS) and 1% penicillin/streptomycin. Cells between Passages 5 and 10 were used in experiments. In all cases, cells were grown until 80–90% confluent and washed three times by rinsing with HBSS before all experiments. HTB‐2 cells were washed using HBSS and Trypsin–EDTA solution, 0.25%, which was added for 10 min, and centrifuged for 10 min at 1000 rpm. Cells were then divided and seeded into 48 well plates at 50,000 cells per well. Cells were grown for 24 hrs in basal media. Experiments were conducted as per the below mentioned different conditions:

##### Inducing inflammation by H_2_O_2_ treatment

Cell monolayers in 48 well plates were chemically stripped and inflamed using hydrogen peroxide (1% H_2_O_2_ in basal media) for 1 hr before HA intervention.

##### Inducing inflammation by protamine sulfate treatment

Cell monolayers in 48 well plates were chemically stripped using protamine sulfate (PS) (10 mg/ml) for 1 hr before HA was added.

##### Inducing inflammation by TNFα treatment

Cell monolayers in 48 well plates were inflamed using TNFα (100 ng/ml) for 1 hr before HA was added.

##### Basal cell conditions

Cell monolayers were cultured in 48 well plates and media was replaced with basal media.

##### HA intervention on HTB‐2 cells

Cells were washed with HBSS. Control (Basal media) or HA solutions (1, 3, 9, 15 mg/ml) were added to wells for 2 hrs, and then washed with HBSS and replaced with basal media for 24 hours. Cell supernatants were removed and stored at −20°C for cytokines analysis.

##### Effect of HA intervention on sGAGs secretion

The Blyscan™ GAG assays (Bicolour, UK) were performed as per the manufacturer's instructions. The standard range was from 1.0 to 5 μg/ml.

##### Effect of HA intervention on cell metabolic activity

An alamarBlue™ assay was performed to test the cell (HTB‐2) metabolic activity upon treating with prepared HA solutions. After HA intervention, basal media was removed and cells washed using HBSS, add 1 ml of 10% AlamarBlue to all wells and incubate for 3 hrs at 37°C. A total of 10% alamarBlue from 48 wells transferred to pre‐labelled 96 well plate with additional three wells of HBSS, absorbance was measured at 550 and 595 nm wavelengths and percentage difference for metabolic activity was calculated.

##### Effect of HA intervention on cytokines

IL‐6 and IL‐8 Human ReadySetGo ELISAs (eBioscience, Hatfield, UK) were performed as per the manufacturer's instructions. The IL‐6 standard ranged from 2 to 200 pg/ml with a sensitivity of 2 pg/ml. The IL‐8 standard ranged from 2 to 250 pg/ml with a sensitivity of 2 pg/ml. Monocyte Chemoattractant Protein‐1 (MCP‐1)/CCL2 DuoSet ELISAs' (RnD Systems, UK) were performed as per the manufacturer's instructions. The MCP‐1/CCL2 standard ranged from 15.60 to 1000 pg/ml with a sensitivity of 15.60 pg/ml.

#### 2.8.2 T84 cell experimental conditions for permeability studies

Human colorectal cell line T84 was used to test the permeability by transwell assay. The cells were grown on tissue culture plastic in basal media consisting of DMEM supplemented with 10% FCS iron supplemented and 1% penicillin/streptomycin. Cells between Passages 5 and 10 were used in experiments. In all cases, cells were grown until 70–80% confluent before being using for experiments. T84 cells were washed using HBSS and Trypsin–EDTA solution, 0.25% added for 10 min, centrifuged for 10 min at 1000 rpm. Cells were then divided and seeded into 12 well (0.4 μm pore) polyester membrane inserts at 500,000 cells per well. Cells were grown until a minimum 1000 Ωcm^2^ per well was reached (10–14 days). Resistance was measured using an Epithelial Volt/Ohm (TEER) Meter (WPI, Sarasota, USA). Experiments were conducted under different conditions.

##### T84 cells treatment with protamine sulphate and inflammation

Cell monolayers in 48 well plates were chemically stripped and inflamed using protamine sulphate (10 mg/ml) and TNFα (100 ng/ml) mixture for 1 hr before HA intervention.

##### HA intervention on T84 cell‐transwells for permeability testing

After inducing inflammation on cells for 1 hr, cells were washed with HBSS. Control (Basal media) or HA solutions (1 or 3 mg/ml) were added to wells for 2 hrs and then washed with HBSS and replaced with basal media for 6 hrs. Trans‐epithelial electrical resistance (TEER) and FITC‐Dextran (4 kDa) flux across T84 monolayers were measured over 6 hrs and the apparent permeability coefficient (Papp) of FITC‐Dextran (4 kDa) was calculated. TEER was measured every hour using 4 mm fixed width double electrodes (STX2) connected to EVOM2 Epithelial Volt/Ohm Meter (World Precision Instruments, Sarasota, USA).

### 2.9 Statistical analysis

Treatments were added in duplicate to each plate of wells and each experiment was run on four separate experimental replicates. Il‐6 and Il‐8 Elisa's were run on four replicates; MCP‐1 was run on three of the four experimental replicates, due to a remaining supernatant volume. All data was normalized to the untreated control and comparisons were analyzed by one‐way ANOVA with Dunnett's post‐test. For FITC‐dextran (4 kDa) Papp measurements across monolayers, individual experiments ran in duplicate and each experiment was run on four separate experimental replicates. Comparisons were determined by Tukey post hoc test to compare groups. Data was presented as the mean ± *SD* with statistical significance indicated as *p < 0.05.

## 3 RESULTS

**FIGURE 1:**
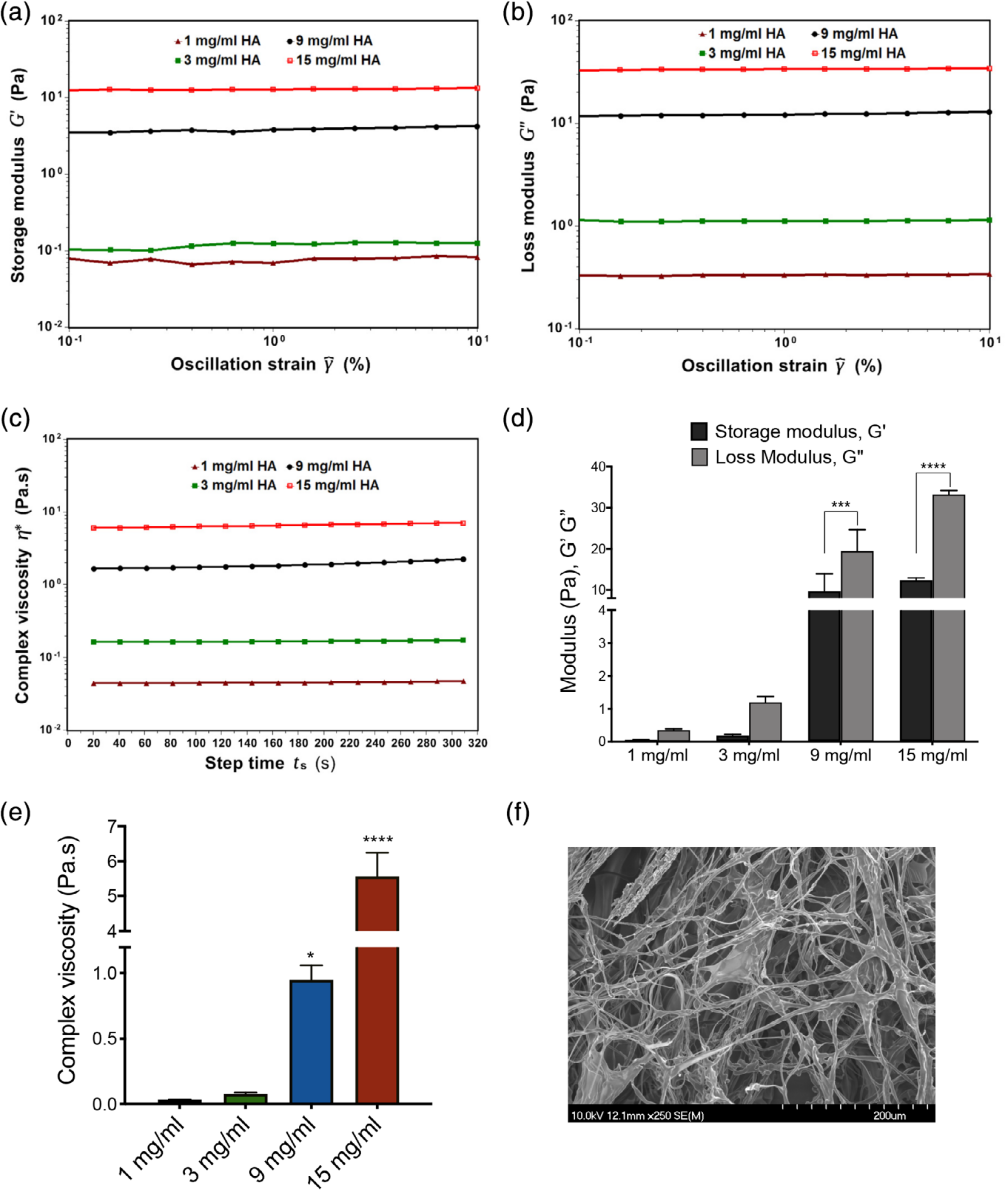
Dynamic oscillatory amplitude, time sweep curves of different naïve HA concentrations effect on viscoelastic properties: (a‐c) Representative viscoelastic curves of storage modulus, G′ (Pa), loss modulus, G″ (Pa), complex viscosities (Pa.s) of different naïve HA solutions. (d) Comparison of G′, G″ of different naïve HA concentration solutions; (e) Comparison of complex viscosities of different HA concentrations. (f) A representative SEM image of freeze‐dried naïve HA solution (3 mg/ml). Data in 1d is represented as mean ± *SD*, Two‐way ANOVA, Sidak's multiple comparisons test. ^***^ *p* < 0.001, ^****^ *p* < 0.0001‐ G′ versus G″ at 9, 15 mg/ml, respectively. Data in 1e is represented as mean ± *SD*, One‐way ANOVA, post hoc Tukey test. ^*^ *p* < 0.05 versus 1 mg/ml; ^****^ *p* < 0.0001 versus 1 mg/ml

Understanding of viscoelastic behavior is critical as the HA‐based system will eventually be administered directly into the bladder via intravesical catheterization. Viscoelastic behavior over a range of HA concentrations (1–15 mg/ml) was assessed to establish the linear viscoelastic properties including modulus and viscosity. The storage modulus and loss modulus (G′ and G″, respectively) and complex viscosity (η*, Pa.s) data showed that, the viscosity is strongly depends on the concentration of HA (Figure 1). The linearity of the modulus G′, G″ under 0.1–10% strain indicated the high stability of the HA system under strain conditions (Figure 1a,b). A maximum value for viscosity of 5.64 ± 0.16 Pa.s was recorded for HA at a concentration of 15 mg/ml (Figure 1c,e). For non‐crosslinked HA (1–15 mg/ml) solution concentrations, G″ was greater than the G′ indicating non‐crosslinked HA system is viscous in nature (Figure 1d). A representative SEM image of freeze‐dried non‐crosslinked HA solution (3 mg/ml) was shown in Figure 1f. Higher concentrations of HA (> 15 mg/ml) were not considered as these concentrations resulted in well‐defined highly viscous/semi solid gels, not suitable for injection through a catheter to the bladder site.

**FIGURE 2:**
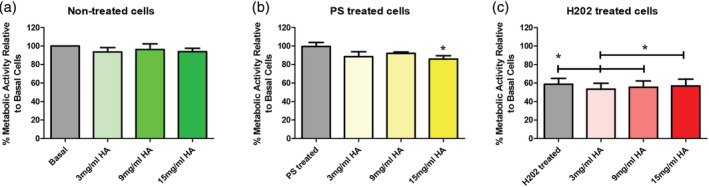
The effect of increasing naïve HA concentration on urothelial cells. (a) Metabolic activity of cells under basal conditions; (b) Metabolic activity of HTB‐2 cells under PS treated conditions; (c) Metabolic activity of HTB‐2 cells under H_2_O_2_ conditions. Data is represented as mean ± *SD*. *N* = 3, *p* values were determined by one‐way ANOVA, post hoc Tukey test and ^*^ *p* < 0.05 than control

**FIGURE 3:**
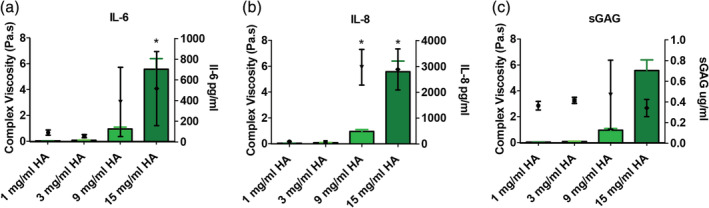
Comparison of the rheological effect (complex viscosity in bars‐green) on IL‐6, IL‐8, and sGAG levels in basal cells (a) Comparison of the complex viscosity of different naïve HA concentration solutions and the effect of viscosity on secreted IL‐6 levels from HTB‐2 cells over 24 hrs; (b) Comparison of the complex viscosity of different naïve HA concentration solutions and the effect of viscosity on secreted IL‐8 levels from HTB‐2 cells over 24 hrs; (c) Comparison of complex viscosity of different naïve HA concentration solutions and the effect of viscosity on secreted sGAG levels from HTB‐2 cells over 24 hrs. * *p* < 0.05 versus 1 mg/ml HA treatment

To test the effect of HA concentration (increasing viscosity) on urothelial cells, three different concentrations were studied on urothelial‐like cells. To replicate the multifactorial disease conditions, cells were pretreated with PS (10 mg/ml) or hydrogen peroxide (H_2_O_2_) (1%) for 1 hr followed by the addition of HA (1, 3, 9, 15 mg/ml) for 2 hrs (Figure 2). It was demonstrated that under control conditions, increasing the HA did not adversely affect the cellular metabolic activity (Figure 2a). When pretreated with PS, only the highest HA concentration (15 mg/ml) demonstrated a significant difference in metabolic activity (Figure 2b). Pretreatment with hydrogen peroxide resulted in a dramatic decrease in the metabolic activity at all HA concentrations tested (Figure 2c). Secretion of sGAG by basal urothelial cells was independent of HA concentration (Figure 3c). Dramatic increases in the levels of interleukin‐6 (IL‐6), 10‐fold increase and a 30‐fold increase in interleukin‐8 (IL‐8) were observed in basal urothelial cells as the concentration of HA increased (Figure 3a,b). Therefore, to avoid HA concentration dependent effects, 1 and 3 mg/ml HA concentrations were used to determine the optimal combination of cross‐linked HA.

**SCHEME 1:**
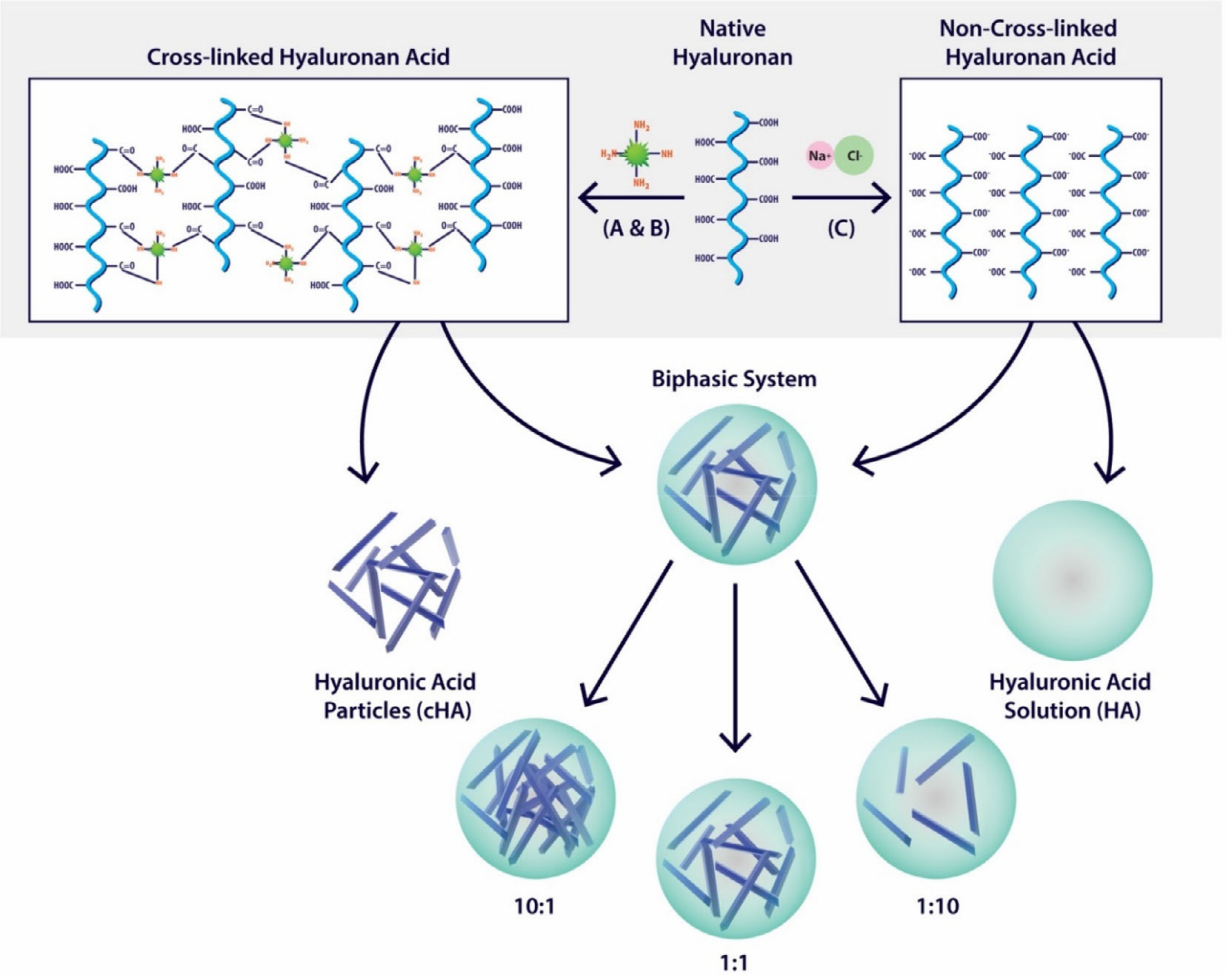
Schematic representation of HA and cross‐linked HA and composition of HA biphasic system. (a) 0.1 M MES Buffer, 20 wt% Na_2_SO_4_ 2 h, RT; (b) DMTMM, overnight, RT; (c) 0.9% NaCl

To determine the effect of cHA compared to naïve/non‐crosslinked HA, a range of combinations of cHA and naïve HA were created (Scheme 1). The synthesized cHA was characterized for purity by NMR. Strong characteristic peaks were observed for acetamide —CH3 of HA at 2.01 ppm and anomeric protons at 4.50 and 4.55 ppm and of O—CH2—CH2 protons of PEG repeating unit at 3.71. Absence of peaks corresponding to DMTMM or its biproducts further confirmed its purity (Figure S3, Figure S4A). Homonuclear correlation spectroscopy (COSY) further revealed no cross‐correlation peaks between protons of PEG and HA (Figure S4B). A time dependent NMR of the polymer in distilled water over a period of 1 week showed no shift in the chemical spectrum of the peaks indicating that the cross‐linked HA is stable at a neutral pH (inset, Figure S4A) Comparing the integrals of NMR peaks at 2.01 ppm (—CH3 of HA) and at 3.22 ppm (—CH2—NH2 of PEG) and considering targeted theoretical cross‐linking of 40% we estimated 24.5% degree of cross‐linking efficiency between HA and PEG.

Carbazole assay was used to estimate the percentage of free carboxylic groups in two samples types: cHA with salt and cHA without salt. These estimations were made using the calibration curve generated for HA 1000 kDa. cHA without salt was estimated to be 37% cross‐linking while samples with salt overestimated the degree of cross‐linking to 66.5%. These observations were consistent with literature reports of salts interfering with carbazole results.^47^, ^48^ Considering the large globular structure of HA and its high molecular weight it is difficult to predict exact degree of cross‐linking but based on these studies it can be estimated to a range between 24.5 and 37% which is less than the theoretically targeted value of 40%.

A 0.9% NaCl content was maintained in the sample to facilitate a direct correlation with current commercially available products administered into the bladder. A known weight of cross‐linked HA was purified by dialysis and lyophilized, and the difference in the weight of the sample before and after dialysis calculated to determine the salt content. The morphology of cross‐linked HA (1 mg/ml) in water (with and without NaCl) was examined using a SEM. At this concentration and post 7 days of incubation, the cross‐linked HA revealed dendritic needle like assembly of approximately 1 μm in thickness. For samples with salt, the presence of salt did not compromise the morphology indicating the stability of the cross‐linked HA under physiological conditions of administration into the bladder (Figure S1). There was no significant difference in the complex viscosity (η*) among HA solutions, cross‐linked HA and a 1:1 ratio of cross‐linked HA to the naïve HA. All systems showed similar viscosities <0.05 Pa.s (Figure S5). Cross‐linked HA was mixed with naïve high molecular weight HA at three different ratios (1:1, 10:1, and 1:10) for further biological evaluation (Figure S2).

**FIGURE 4:**
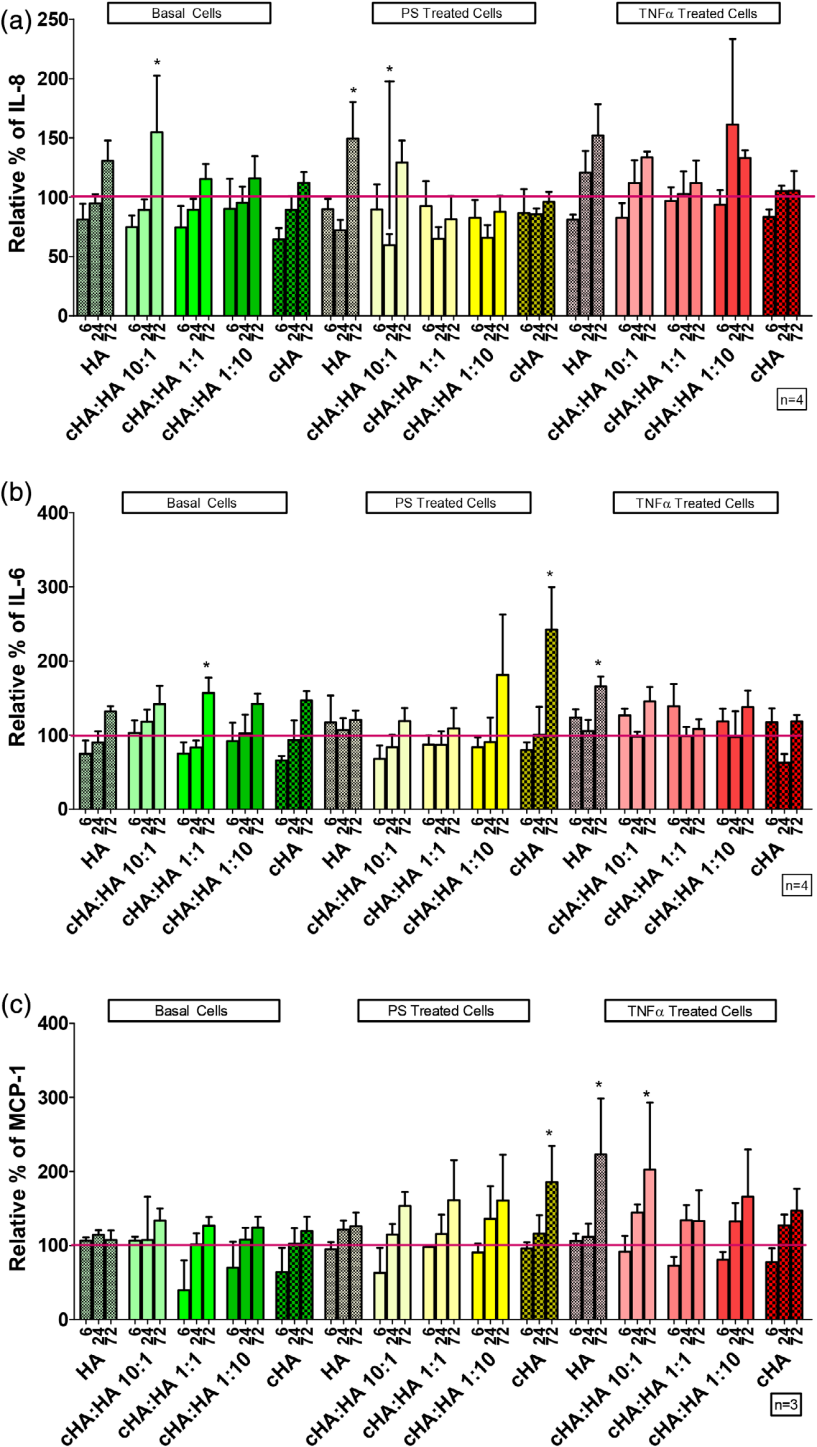
Effect of cHA:HA ratios at 1 mg/ml on inflammatory cytokines. (a) Comparison of different ratios of cHA:HA at 1 mg/ml on secreted IL‐8 levels compared to control (red line) from HTB‐2 cells over 6, 24, and 72 hrs. (b) Comparison of different ratios of cHA:HA at 1 mg/ml on secreted IL‐6 compared to control (red line) levels from HTB‐2 cells over 6, 24, and 72 hrs. (c) Comparison of different ratios of cHA:HA at 1 mg/ml on secreted MCP‐1 levels compared to control (red line) from HTB‐2 cells over 6, 24, and 72 hours. Statistical significance indicated as *p < 0.05 compared to control cells at same time point

The cross‐linked HA to naïve HA ratios were examined at two concentrations, 1 mg/ml HA and 3 mg/ml HA for their effect on the expression on IL‐6, IL‐8, and MCP‐1 on basal cells pre‐treated with TNFα (100 ng/ml) and PS (10 mg/ml) to mimic differing disease pathologies. The different ratios of cross‐linked HA to naïve HA and depending on pre‐treatment, the varying ratios caused differential secretion of IL‐6 (Figure 4a), IL‐8 (Figure 4b) and MCP‐1 (Figure 4c). The effect of the varying ratios of cross‐linked HA and naïve HA (cHA:HA) under basal conditions, revealed that the 10:1 ratio of cHA:HA after 72 hrs significantly increased IL‐8 levels (54%) compared to the control. In PS treatment conditions, treatment with 10:1 ratio of cHA:HA after 24 hrs significantly decreased the IL‐8 levels (41%) compared to HA alone, however it was not observed at 72 hours. Treatment with naïve HA resulted in a significant increase (49%) of IL‐8 levels compared to control (Figure 4a). Under normal basal conditions, treatment with 1:1 ratio of cHA:HA after 72 hrs significantly increased IL‐6 levels (56%) compared to the control (Figure 4b). For PS treatment conditions, treatment with cHA significantly increased the IL‐6 levels (142%) compared to control after 72 hrs. On the other hand, TNFα pre‐treated conditions, it was observed that the naïve HA significantly increased IL‐6 levels (65%) at 72 hours compared to control (Figure 4b). There was no difference in MCP‐1 levels under normal basal conditions regardless of treatment. For PS treatment conditions treatment with cHA significantly increased MCP‐1 levels (85%) after 72 hrs compared to control. Under TNFα pre‐treated conditions, it was observed that, after 72 hrs, the naïve HA and the treatment with 10:1 ratio of cHA:HA significantly increased MCP‐1 levels (222 and 202%, respectively) (Figure 4(C)).

**FIGURE 5:**
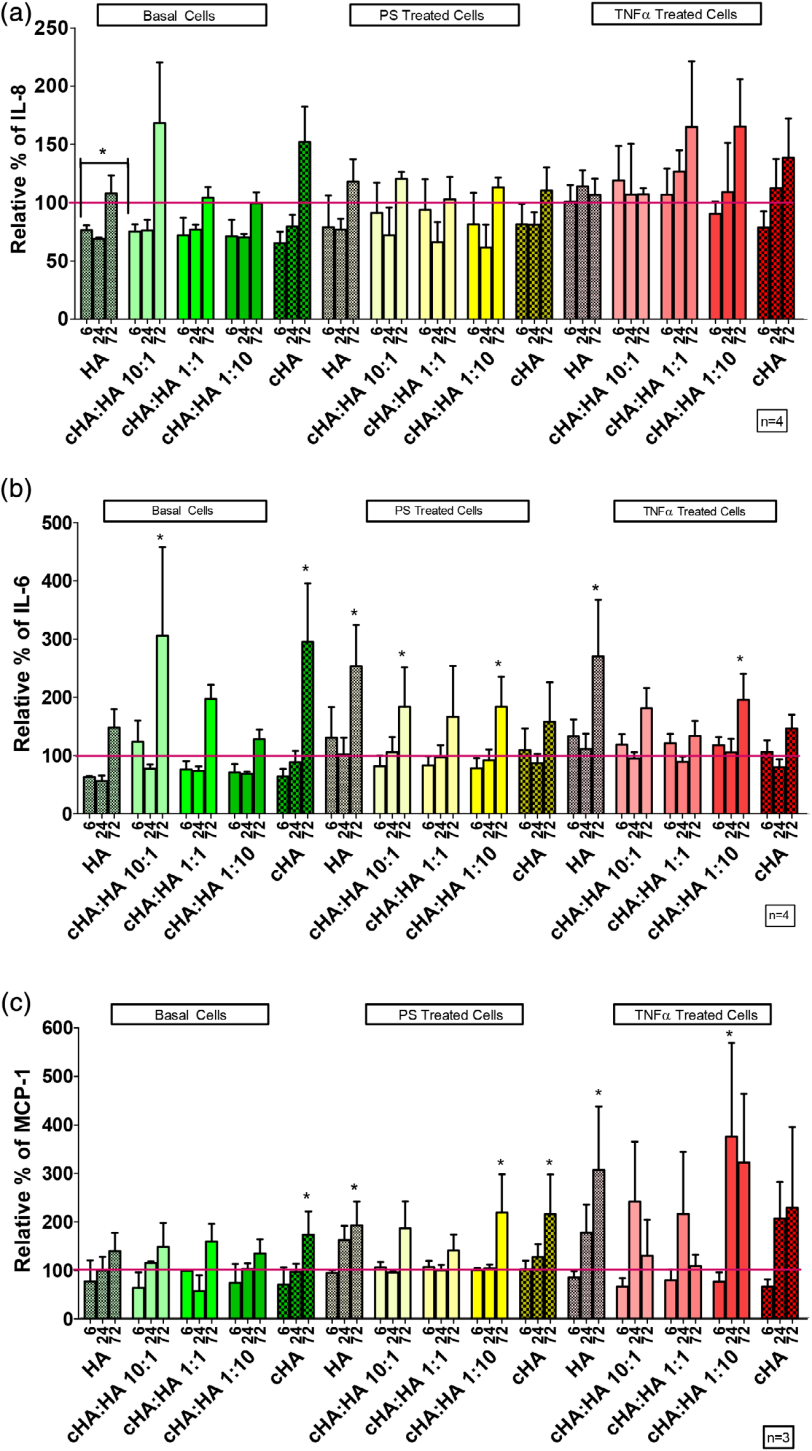
The effect of cHA:HA ratios at 3 mg/ml on inflammatory cytokines. (a) Comparison of different ratios of cHA:HA at 3 mg/ml on secreted IL‐8 levels compared to control (red line) from HTB‐2 cells over 6, 24, and 72 hrs. (b) Comparison of different ratios of cHA:HA at 3 mg/ml on secreted IL‐6 levels compared to control (red line) from HTB‐2 cells over 6,24 and 72 hours. (c) Comparison of different ratios of cHA:HA at 3 mg/ml on secreted MCP‐1 compared to control (red line) levels from HTB‐2 cells over 6, 24, and 72 hrs. Statistical significance indicated as *p < 0.05 compared to control cells at same time point

Pre‐treatment with 3 mg/ml cHA:HA treatments resulted in the differential secretion of IL‐6 (Figure 5a), IL‐8 (Figure 5b) and MCP‐1 (Figure 5c). Under normal basal conditions naïve HA and a 10:1 ratio of cHA:HA at 6 hrs significantly decreased IL‐8 levels (24 and 25%, respectively) compared to the control. However, at 24 and 72 hrs this effect had disappeared. Under PS pre‐treated conditions and TNFα pre‐treated conditions there was no significant changes in IL‐8 levels compared to control (Figure 5a). Under basal treatment conditions the 10:1 ratio of cHA:HA and HA alone significantly increased IL‐6 levels (205 and 295%, respectively) at 72 hrs compared to the control. Under PS‐treated conditions the naïve HA, 10:1 ratio 1:10 ratio of cHA:HA significantly increased the IL‐6 levels (53, 83, and 83%, respectively) at 72 hrs compared to control. Under TNFα pre‐treated conditions, it was observed that naïve HA and 1:10 ratio of cHA:HA significantly increased IL‐6 (170 and 95%, respectively) at 72 hrs (Figure 5b). Similar to as seen in Figure 4c, under normal basal conditions, MCP‐1 levels were not altered by treatment at 3 mg/ml. However, under PS treatment conditions naïve HA, 10:1 ratio, 1:10 ratio and cHA alone significantly increased the MCP‐1 levels (92, 86, 119 and 115%, respectively) after 72 hrs compared to control, while 1:1 ratio increased MP‐1 levels 61%. Under TNFα treatment conditions, naïve HA and a 1:10 ratio of cHA:HA significantly increased MCP‐1 levels (207 and 275%, respectively) after 72 hrs compared to control (Figure 5c).

**FIGURE 6:**
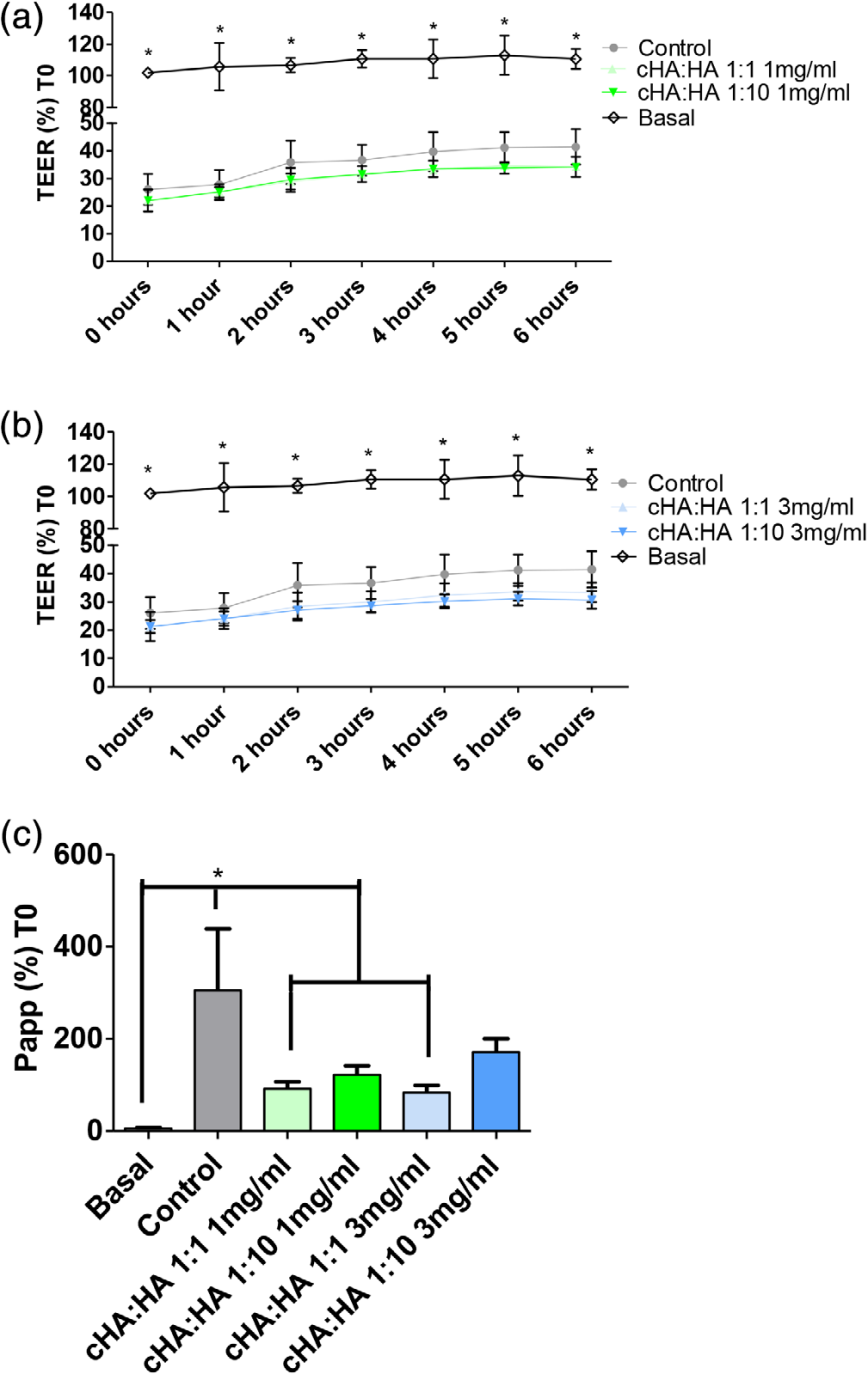
The effect of different ratios of cross‐linked HA to naïve HA on permeability. (a) Measurement of TEER on T84 cells treated with different ratios of cross‐linked HA to naïve HA at a HA concentration of 1 mg/ml HA on T84 cells pretreated with protamine sulfate and TNFα mixture (b) Measurement of TEER on T84 cells treated with different ratios of cross‐linked HA to naïve HA at a HA concentration of 3 mg/ml HA on T84 cells pretreated with protamine sulfate and TNFα mixture. (c) Measurement of Papp on the effect of different ratios of cross‐linked HA to naïve HA at both 1 and 3 mg/ml over 6 hrs on T84 cells pretreated with protamine sulfate and TNFα mixture *p < 0.05 compared to basal cells at same time point. (Basal‐healthy/uninflamed cells; Control‐protamine sulfate and TNFα mixture pretreated), *N* = 3

On the other hand, the HTB‐2 cells did not form a stable measurable resistance; (data not shown) henceforth, colon epithelial cells (T84) are used to mimic an in vitro model of a tight epithelium monolayer system and were grown on transwell membranes until they had a measurable TEER greater than 1000Ωcm.^2^ TEER was measured hourly over 6 hrs on cells pre‐treated with PS and TNFα (inflammation‐1 hr) followed by treatment with 1 mg/ml (1:1 and 1:10 ratio cHA:HA) (Figure 6a) and 3 mg/ml (1:1 and 1:10 ratio cHA:HA) (Figure 6b) with control cells treated with normal media. It was observed that the monolayers that received the aggressive pre‐treatment of PS and TNFα mixture had higher Papp compared to those control cells. In this combination disease model, treatments with a 1:1 ratio of cHA:HA at both 1 and 3 mg/ml HA concentrations significantly decreased the Papp of T84 cell monolayers, without altering the TEER (Figure 6c).

## 4 DISCUSSION

Currently available intravesical sGAG/GAG treatments are available in a range of concentrations, 0.8, 1.6, and 2.4%.^49^, ^50^ There is sufficient evidence to show that the effect of viscosity correlates with increased HA concentration (Figure 1). When examining the effect of HA concentration in a urothelial cell monolayer without a consensus on the exact conditions of mimicking IC pathogenesis can be challenging. To mimic barrier damage to the urothelium, two approaches were used, hydrogen peroxide (1%) and PS (10 mg/ml) for 1 hr alongside a non‐treated basal media control. Hydrogen peroxide is oxidizing agent that increases the permeability of cell membranes and PS is a highly cationic peptide that increases the permeability of tissue.^51^, ^52^, ^53^

Under normal basal conditions, it was observed that there was no change in metabolic activity (Figure 2a); however, when the cells are stressed by PS, the highest HA concentration significantly decreased the cellular metabolic activity (Figure 2b). Under extremely stressful conditions where cells were pre‐treated with hydrogen peroxide, regardless of the HA concentration; the cell metabolic activity was significantly decreased. This is due to the severity of the hydrogen peroxide pretreatment, which also decreased the metabolic activity of control cells by 40% and may be killing the cells (Figure 2c). In order to understand the effect of increasing HA concentration on IC disease markers, IL‐6, IL‐8, and sGAG levels were examined (Figure 3). IL‐8 is associated with increased mast cells in the bladder.^54^, ^55^ IL‐6 is an inflammatory factor that has been found to be independently correlated with C‐reactive protein and IL‐8 in the serum of IC patients.^56^ MCP‐1 has been reported at elevated levels in preclinical models, which replicate the disease symptoms. The urine of IC patients has also been reported to contain higher levels of sGAG along with IL‐6, IL‐8, and MCP‐1.^16^ These factors have been associated with the disease status of patients and have been shown to decline in some patients who respond to therapy. It was demonstrated (Figure 3) that 9 and 15 mg/ml HA dramatically increased (10–30‐fold) the secretion of IL‐6, IL‐8, and sGAG. Therefore, to avoid increasing these factors, we used 1 and 3 mg/ml HA concentrations to test the ratios of cHA and naïve HA. This concentration range is similar to the concentration using in commercially available treatments.

Cross‐linking of HA was performed to increase the degree of polymerization, to decrease the permeability of HA without increasing inflammation. The length of HA inversely correlates with the amount of HA absorbed, with longer chain HA decreasing the permeation of the tissue by HA.^57^ Short chain oligosaccharides can have a substantially different effect on immune response and permeability.^58^, ^59^ The cross‐linking of HA created small (1 μm width) dendritic like morphology (Figure S1). The cHA was tested in various ratios with naïve HA, to determine the effect of the cross‐linking and the degree of polymerisation (Scheme 1). The cross‐linking of HA was performed using DMTMM and PEG. After purification of the construct, DMTMM was not observed in the final reaction mixture (Figure S4). The PEG signal was consistently observed over 7 days in distilled water. The NMR spectra showed no change in the chemical shift of the peaks suggesting the absence of non‐covalent interactions and the stability of the cross‐linked HA at a neutral pH, which indicates that there will be limited degradation to the cross‐linked HA the testing time period.

There are several theories on the etiology and pathogenesis of IC. One of the theories is that IC is inflammation driven and an inflammatory cascade leads to a change in the permeability of the urothelium and the damage from urinary solutes infiltrating the urothelium leads to a cycle of inflammation and urothelium damage.^7^, ^60^ A more recent hypothesis states that a change in permeability leads to urine solutes infiltrating the urothelium, leading to inflammation, pain and a cycle of inflammation and urothelium damage.^61^ However, with no clear evidence for the etiology of IC, the treatments were tested in several disease‐like conditions (Figure S2). As stated above, with no clear etiology, the disease state was broken into separate areas of testing. Our initial testing using hydrogen peroxide to cause inflammation and increased permeability (Figure 2c) could not be used due to a large decrease in metabolic activity. Instead to emulate the disease state, TNFα was added to simulate inflammation and to test the alternative hypothesis PS pretreatment was used to change the urothelial cell permeability. As a control, basal media conditions represent the healthy urothelium. To understand the effect of different formulations on known disease aspects, inflammation (Figures 4 and 5) and permeability (Figure 6) were examined to create a multifaceted picture of the potential of cHA to treat IC.

Cross‐linked HA alone at 1 mg/ml HA concentration in PS treated cells increased MCP‐1 and IL‐6, while cross‐linked HA alone at 3 mg/ml HA concentration increased MCP‐1 levels (Figures 4 and 5). Naïve HA at 1 mg/ml concentration increased IL‐6 and MCP‐1 in inflamed TNFα treated cells. Naïve HA at 3 mg/ml concentration in PS treated and TNFα treated cells increased the expression of IL‐6. However, in contrast to the increased levels observed in several treatment groups, the 1:1 ratio of cross‐linked HA to naïve HA at a 1 mg/ml or 3 mg/ml HA concentration did not increase the levels of IL‐8, IL‐6 or MCP‐1.

**FIGURE 7:**
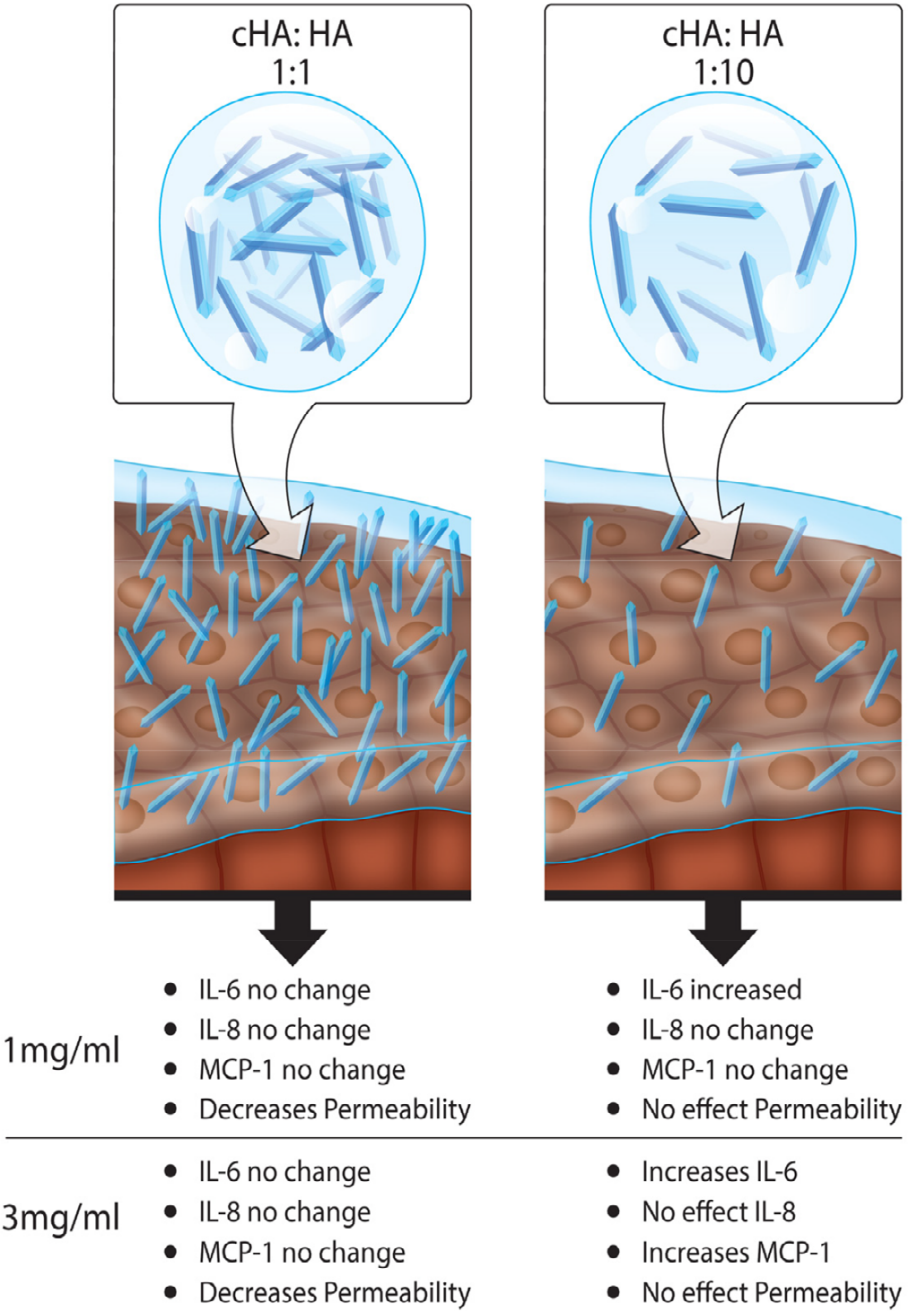
The effects of the different ratios and concentrations on the key indicators of IC. Cross‐linking HA decreased the apparent permeability, without altering the disease conditions at a 1:1 ratio of cross‐linked HA to naïve HA at both 1 and 3 mg/ml HA concentrations

This combination treatment was then tested to examine its effect in a transwell epithelium model of barrier permeability. Normal healthy human urothelium is a highly impermeable effective epithelium, forming a high resistance (>2,500 Ωcm^2^), low flux barrier, preventing the invasion of urinary solutes into the underlying tissues. For permeability testing, we examined whether cross‐linked HA would improve the barrier function in a cell monolayer system. T84 cells were grown on transwell permeable membranes, to have a resistance of >1000Ωcm^2^. At >1000Ωcm^2^, the transwell cells were stable and a measurable change in permeability was able to be measured. This reproducible low permeability monolayer is a test bed for examining barrier changes. The measurement of TEER is a reliable method to examine the integrity and by extension the permeability of a monolayer of cells. The Papp is a method used to examine drug absorption, it examines the migration of a particular sized molecule across a monolayer, and variations in TEER usually correlate with changes in Papp.^62^, ^63^ However, treatment with a 1:1 ratio of cross‐linked HA to naïve HA at a 1 and 3 mg/ml HA concentration decreased the apparent permeability of the cell monolayer to 4 kDa molecules without a corresponding increase in TEER (Figure 6). TEER remained low in the cells, while 2 hrs of treatment with a 1:1 ratio of cHA:HA had an increased barrier effect as observed by the significant decrease in Papp. A physiochemical barrier effect from the increased chain length in the 1:1 ratio of cHA:HA leads to a decrease in Papp without an alteration in the expression in TEER. In Figure 7 the overall effects of the different ratios and concentrations effects on the key indicators of IC is discussed. It has been observed that the cross‐linking of HA decreases the apparent permeability, without altering the disease conditions at a 1:1 ratio of cross‐linked HA to naïve HA at both 1 and 3 mg/mlL HA concentrations.

## 5 CONCLUSIONS

We have successfully demonstrated the engineering of a biphasic system developed by combining cHA and naive HA solution to reduce inflammation and permeability in the in vitro interstitial cystitis model. In our study, we observed that increasing the concentration of naïve HA solutions (1–15 mg/ml), there was corresponding increase in viscosity, which increased the secretion of inflammatory cytokines (IL‐6 and IL‐8). These results were deciding factor to determine the concentration of naïve HA for cross‐linking. The cross‐linking was successfully performed on (1 and 3 mg of HA) by using 4‐arm PEG chemistry and degree of cross‐linking was confirmed by carbazole assay. The biphasic system reduced permeability, while at the same time ensured that there is no undue effect on cytokines or an adverse increase in the viscosity of the solution. Our study suggests that the ratio of 1:1 (cHA:HA) of 1 and 3 mg/ml formulations has proven the ability to decrease the permeability and alter inflammatory cytokines levels in in vitro model of IC. This developed biphasic system holds promise in augmenting the protective element of the bladder and provides a permissive healing environment for the urothelium. The developed biphasic system of HA formulation is a promising candidate to modify the GAG‐based therapy in IC. Further studies are needed to investigate the therapeutic efficacy in a suitable in vivo cystitis model.

## CONFLICT OF INTEREST

The authors declare no potential conflict of interest.

## AUTHOR CONTRIBUTIONS

The manuscript was written through contributions of all authors. All authors have given approval to the final version of the manuscript.

**Abbreviations**
cHA: crosslinked hyaluronic acid
COSY: homonuclear correlation spectroscopy
CS: chondroitin sulfate
DMTMM: (4‐[4,6‐dimethoxy‐1,3,5‐triazin‐2‐yl]‐4‐methyl‐morpholinium chloride)
EtOH: ethanol
EDTA: ethylenediaminetetraacetic acid
FBS: fetal bovine serum
FCS: fetal calf serum
FITC: fluorescein isothiocyanate
G′: storage module
G″: loss modulus
GAG: gylcosaminoglycans
HA: hyaluronic Acid
HBSS: hanks balanced salt solution
HSQC: heteronuclear single quantum correlation
IC: interstitial cystitis
IL‐6: interleukin six
IL‐8: interleukin eight
MCP‐1: monocyte chemoattractant protein‐1
NaCl: sodium chloride
NMR: nuclear magnetic resonance spectroscopy
Papp: apparent permeability
PEG: polyethylene glycol
PS: protamine sulfate
sGAG: sulfated gylcosaminoglycans
TEER: trans epithelial electrical resistance
TNFα: tumor necrosis factor alpha

## Supporting information


**Figure S1** The morphology of cross‐linked hyaluronic acid (cHA) (1 mg/mL) as determined by SEM.
**Figure S2:** Concentration testing process to determine appropriate concentrations for ratio testing.
**Figure S3:** NMR spectra of cHA for which the peak report was generated
**Figure S4:** NMR of cross‐linked HA in distilled water (A), overlay of NMR for 7 days (inset); (B) Homonuclear correlation spectroscopy (COSY) of the cross‐linked HA polymer; (C) Heteronuclear single quantum correlation (HSQC) spectrum of cross‐linked HA
**Figure S5:** HA solution, cross‐linked‐HA (cHA) and the effect of a 1:1 ratio of cHA) to Naïve HA concentration; Solution of HA (3 mg/mL), cHA (3 mg/mL) and combination (HA: cHA = 1:1) effect on complex viscosity, η* (Pa.s)
(File type PDF)
**Table S1:** NMR peak report of cHA
