# Supplementary material for: A high molecular weight hyaluronic acid biphasic dispersion as potential therapeutics for interstitial cystitis
Source: J Biomed Mater Res B Appl Biomater. 2020 Oct 26;109(6):864–76. doi: 10.1002/jbm.b.34751 (PMC8246519; doi:10.1002/jbm.b.34751)
Supplement: Supplementary file 1 — Figure S1 The morphology of cross‐linked hyaluronic acid (cHA) (1 mg/mL) as determined by SEM. Figure S2: Concentration testing process to determine appropriate concentrations for ratio testing. Figure S3: NMR spectra of cHA for which the peak report was generated Figure S4: NMR of cross‐linked HA in distilled water (A), overlay of NMR for 7 days (inset); (B) Homonuclear correlation spectroscopy (COSY) of the cross‐linked HA polymer; (C) Heteronuclear single quantum correlation (HSQC) spectrum of cross‐linked HA Figure S5: HA solution, cross‐linked‐HA (cHA) and the effect of a 1:1 ratio of cHA) to Naïve HA concentration; Solution of HA (3 mg/mL), cHA (3 mg/mL) and combination (HA: cHA = 1:1) effect on complex viscosity, η* (Pa.s) (File type PDF) Table S1: NMR peak report of cHA [file JBM-109-864-s001.docx]

**Supplementary information for publication**

**A High Molecular Weight Hyaluronic Acid Biphasic Dispersion as Potential Therapeutics for Interstitial Cystitis**

Peadar R Rooney^1^, Vijaya Krishna K.^1^, Niranjan G. Kotla^1^, Ana Benito^2^, Damien Dupin^2^, Iraida Loinaz^2^, Leo R. Quinlan^1,3^, Yury Rochev^1,4^, Abhay Pandit^1^*

^1^ CÚRAM, SFI Research Centre for Medical Devices, National University of Ireland Galway, Ireland

^2^CIDETEC, Parque Científicoy Tecnológico de Gipuzkoa, San Sebastián, Spain

^3^Physiology, School of Medicine, National University of Ireland Galway, Ireland

^4^Sechenov First Moscow State Medical University, Institute for Regenerative Medicine, Moscow, Russian Federation

Total **five figures** and a **table**


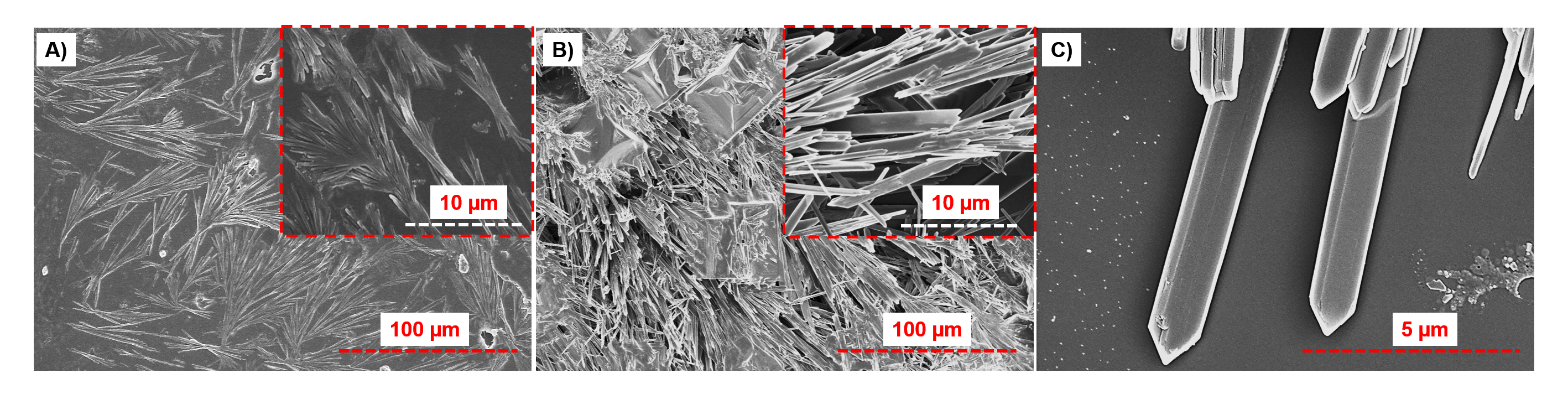


**Figure S1:** The morphology of cross-linked hyaluronan (cHA) (1 mg/ml) post 7 days post incubation as determined by SEM: (A) cHA without salt; (B) dendritic pattern of the polymer (inset), cHA with salt; (C) dendritic pattern of the polymer with salt crystals as points of nucleation (inset), a zoomed image showing the needle like morphology of the polymer.

**Figure S2:** Concentration testing process to determine appropriate concentrations for ratio testing. The selected concentrations were included in the design of the cross-linked HA and naïve HA in different ratios and examined its effect on interstitial cystitis disease markers. In the final stage, the optimized ratios and concentrations were examined for their effect on a model of urothelium barrier permeability.


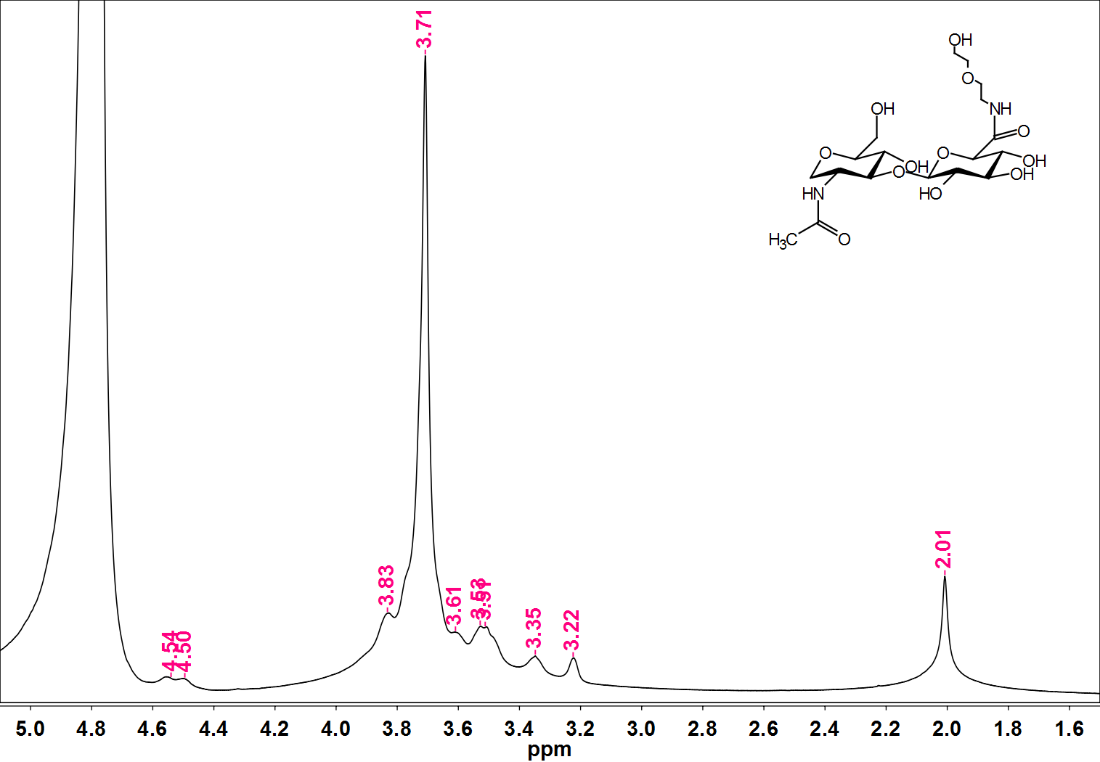


**Figure S3**: NMR spectra of cHA for which the peak report was generated to calculate degree of crosslinking.


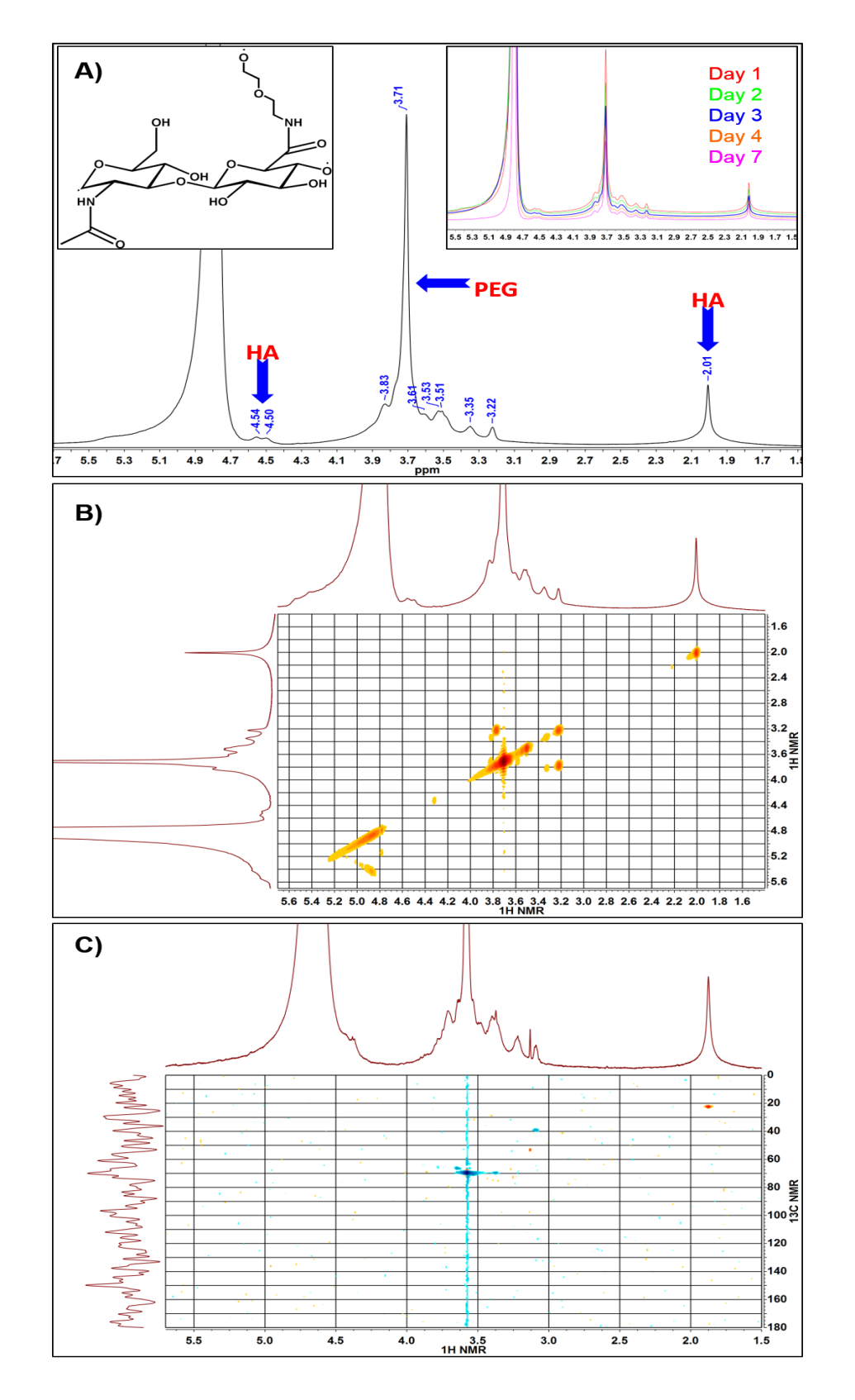


**Figure S4:** NMR of cross-linked HA in distilled water (A), overlay of NMR for 7 days (inset); (B) Homonuclear correlation spectroscopy (COSY) of the cross-linked HA polymer; (C) Heteronuclear single quantum correlation (HSQC) spectrum of cross-linked HA.


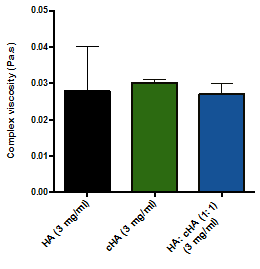


**Figure S5:** HA solution, cross-linked-HA (cHA) and the effect of a 1:1 ratio of cHA) to Naïve HA concentration; Solution of HA (3 mg/ml), cHA (3 mg/ml) and combination (HA: cHA=1:1) effect on complex viscosity, η* (Pa.s).

**Table S1**: NMR peak report of cHA.

| **Peaks** | | | | |
| --- | --- | --- | --- | --- |
| **Number of peaks** | **ppm** | **Intensity** | **Width** | **Area** |
| 1 | 4.54 | 13.5 | 0.88 | 126.98 |
| 2 | 4.50 | 13.4 | 1.47 | 210.27 |
| 3 | 3.83 | 24.7 | 44.02 | 11635.41 |
| 4 | 3.71 | 121.0 | 21.39 | 27693.38 |
| 5 | 3.61 | 21.4 | 0.88 | 201.83 |
| 6 | 3.53 | 22.4 | 21.86 | 5251.10 |
| 7 | 3.51 | 22.2 | 2.05 | 488.83 |
| 8 | 3.35 | 17.4 | 3.37 | 627.02 |
| 9 | 3.22 | 17.0 | 15.11 | 2745.47 |
| 10 | 2.01 | 31.1 | 49.92 | 16625.17 |

**Protocol for carbazole assay** **^1,2^**

**Estimation by Carbazole assay:** Carbazole assay was also performed on the samples to determine the degree of cross-linking. Samples with and without salt were used for the assay. Degree of cross-linking was estimated to 37% using carbazole assay.

**Reagent A** (25 mM sodium tetraborate in sulfuric acid). Dissolve 0.95 g of sodium tetraborate decahydrate in 2 mL of hot water and add 98 mL of ice-cold concentrated sulfuric acid carefully with stirring.

**Reagent B** (0.125% wt/vol carbazole in absolute ethanol). Dissolve 125 mg of carbazole (recrystallized from ethanol) in 100 ml of absolute ethanol to give a stable reagent.

**Assay protocol:** Calibration curve for the carbazole assay was prepared by using HA (60 and 1000 kDa.). Add, separately, aliquots of 25 μL of HA (containing 0, 0.5, 1.0, 1.5, 2.0 and 2.5 μg) to a 96-well microtiter plate in a refrigerated chamber. Add aliquots of 25 μL of cHA sample (dilutions were prepared from a 5 mg/mL stock solution) to the microtiter plate in the refrigerated chamber. Add 150 μl of ice-cold Reagent A to the microtiter plate with mixing and cooling in the refrigerated chamber.4. Heat the mixture at 100 °C for 10 min in an oven (temperature setting at 100 °C). Cool the reaction rapidly in the refrigerated chamber. Add 5 μL of Reagent B and mix well. Reheat at 100 °C for 15 min in the oven. Cool the reaction rapidly in the refrigerated chamber. Determine the absorbance at 525 nm with a 96-well microtiter plate reader.

References:

1. Bitter T, Muir HM. A modified uronic acid carbazole reaction. Anal. Biochem. 1962;4:330-34.

2. Volpi N, Linhardt RJ. High-performance liquid chromatography-mass spectrometry for mapping and sequencing glycosaminoglycan-derived oligosaccharides. Nat. Protoc. 2010;5(6):993-1004.
